# Disclosure of Spousal Death to Patients with Dementia: Attitude and Actual Behavior of Care Managers

**DOI:** 10.3390/ejihpe13020031

**Published:** 2023-02-08

**Authors:** Hisashi Kato, Eisuke Nakazawa, Katsumi Mori, Akira Akabayashi

**Affiliations:** 1 Department of Biomedical Ethics, Faculty of Medicine, University of Tokyo, Tokyo 1130033, Japan; 2 General Practice Department, Chichibu Municipal Hospital, Chichibu 3680025, Japan; 3 Division of Medical Ethics, School of Medicine, New York University, New York, NY 10016, USA

**Keywords:** disclosure of spousal death, dementia, truth telling

## Abstract

As the number of dementia patients increases, there is a need to protect patients’ right to know. However, in reality, there are cases in Japan where spouses’ deaths are concealed from patients. We conducted a questionnaire survey of care managers (CMs) to obtain their attitude and actual behavior regarding the disclosure of a spouse’s death to patients with dementia. A self-administered, anonymous questionnaire survey was implemented at academic meetings attended by CMs from March to December 2019, inquiring about experiences with spousal deaths of patients with dementia, disclosure rates, behavioral and psychological symptoms of dementia, and depression. Over 80% had experienced the spousal death of a patient with dementia; the percentage of CMs who had implemented the disclosures varied widely. About 18% had experienced worsening behavioral and psychological symptoms of dementia (BPSD), and 26% had worsening depression as a result of the disclosure. About 83% of respondents were positive about disclosure, but about 44% did so less than 50% of the time. This study is the first to reveal the current state of CMs’ policies and behaviors regarding the disclosure of spousal death to patients with dementia in Japan. Family members’ wishes and the possibility of BPSD put a relatively large number of caregivers in a dilemma regarding disclosure.

## 1. Introduction

Currently, there are approximately 50 million people with dementia worldwide, and the number is expected to increase to 150 million by 2050 [1]. Human rights issues related to people with dementia are therefore attracting attention [2]. One ethical question that arises is whether care workers should lie to people with dementia [3,4,5,6,7,8]; for example, what should a care worker do if a dementia patient forgets about the death of a close relative [9]? In Japan, there are cases where those with dementia are not informed that their spouse has died. Thus, a careful examination regarding whether this practice is appropriate must be conducted [10].

There are several benefits of knowing that a spouse has died. First, there is a right to information. This leads to treating people with dementia as respectful and autonomous personalities. Second, people with dementia can attend the funeral if they wish. This would aid the adjustment to loss and thereby mitigate confusion about spousal absence. Conversely, there are disadvantages to disclosing a spouse’s death to people with dementia, which may be the most painful event in their lives. People with dementia have impaired memories and can forget things, even if they are informed. In that case, the person may experience distress about the death of their spouse repeatedly. Additionally, the disclosure may cause psychological stress and worsen the behavioral and psychological symptoms of dementia (BPSD), the symptoms of disturbed perception, the thought content, the mood, or the behavior that frequently occur in patients with dementia [11,12,13]. In addition, people with dementia are at a high risk for depression [14,15,16], and the information about their spouse’s death could cause them to develop depression and develop suicidal ideation [17,18]. Due to these concerns, the family may not wish to disclose the death of a spouse to the person with dementia. In these situations, care workers for an individual with dementia often hesitate to persuade their family to disclose the information about the spouse’s death. This problem should be addressed as a human rights issue for people with dementia, especially as the number of dementia patients is increasing rapidly. While disclosing spousal death may be justified from the perspective of respecting the autonomy and the right to know of the patient with dementia, it cannot be justified from the principle of non-maleficence. This forms an ethical dilemma in which the two ethical principles and values of respect for autonomy and non-maleficence are incompatible.

In Japan, a long-term care system centered on care managers (CM) has been established. CM is a national certification under the Long-Term Care Insurance Act [19]. CMs make care plans for individuals over 65 based on the Long-Term Care Insurance Law and support their lives at home and in institutions. In general, people with dementia have CMs assigned to them. The CM visits their home at least once a month and consults with them and their family about any problems with their care. Therefore, when the spouse of a person with dementia dies, the family often consults with the CM before telling the individual with dementia [20]. The CM is, therefore, the key person who decides whether or not a spousal death is disclosed to a person with dementia. Studies have examined how to counsel people with dementia about the death of their close relatives [21] and the legitimacy of lying to dementia patients in clinical practice [22,23].

In this study, we conducted cross-sectional research targeting care managers. The purpose of this study is to clarify CMs’ attitudes toward the disclosure of a spouse’s death for people with dementia and to investigate related factors. This survey is considered the first step toward formulating guidelines to address the disclosure issues that the care environment for dementia patients, including those requiring care management, is expected to face. The establishment of such guidelines can lead to the creation of a better care environment for dementia patients, including requiring care management, and thereby improve their well-being in the future.

## 2. Methods

### 2.1. Questionnaire

The research was conducted with CMs who voluntarily participated in an academic conference and workshop in Japan from March 2019 to December 2019. Research participation request forms and questionnaires were distributed to the CMs and were collected after they were completed. The CMs were informed that there were no benefits or disadvantages based on the completion of the research and that answers were completely anonymous.

A draft of a questionnaire was developed in consultation with ethics experts based on the experience of the clinicians. In order to refine the draft of the questionnaire, a pilot study was conducted with three clinicians and 24 CMs. The pilot study was conducted to obtain expert opinions on the content validity, and the results were used only to improve the questionnaire items.

Thus, the questionnaire items included demographic information, such as sex, age, years of service, and qualifications (senior CM, certified CM). Subsequently, participants were asked about their experience with spousal death for people with dementia, including the number of cases they had experienced, the percentage of cases they had disclosed (0–100%, in 10% increments), and whether they had experienced cases of worsening BPSD and depression after disclosure. These factors were most frequently expressed as concerns by the care managers in the pilot survey. Regarding the worsening of BPSD and the development of depression after disclosure, we defined these as adverse events that affected care, regardless of whether or not there was a medical diagnosis. Ensuingly, we investigated their attitude toward spousal death disclosure (should be disclosed, better to disclose, no need to disclose, better to not disclose) and asked what they consider to be a high priority in considering the issue of spousal death disclosure for people with dementia. We also inquired how far along in the progression of dementia they would prefer to be disclosed, i.e., whether there is a limit in dementia severity after which they would not disclose, based on the functional assessment staging of Alzheimer’s disease (FAST) classification [24]. 

Furthermore, we investigated what CMs advise for families that do not wish to disclose spousal death (non-intervention, reconsideration of disclosure, recommend disclosing) as well as what actions they themselves take in this scenario (consult with a colleague’s care manager and an attending physician, hold a multidisciplinary conference, do not consult).

### 2.2. Analysis

First, descriptive statistics on the participants’ basic attributes and experiences with spousal deaths of patients with dementia, as well as their attitude and actual behavior regarding disclosure, were calculated.

Next, according to their attitude toward spousal death disclosure, the participants were classified into two groups: a pro-disclosure group for “should be disclosed” and “better to disclose” and an anti-disclosure group for “no need to disclose” and “better to not disclose.” Similarly, participants were classified into two groups, a low disclosure rate group and high disclosure rate group, based on the median value of the ratio of disclosure implementation. The consistency between the attitude toward disclosure and the disclosure rate was examined for those with an experience in cases involving the spousal death of people with dementia.

Finally, Fisher’s exact test was conducted to examine the difference between the group who thought they should be disclosed but were not disclosed and the group who thought they should be disclosed and did so, focusing on the pro-disclosure group.

Statistical analysis was performed using SAS version 9.4 (SAS Institute Inc., Cary, NC, USA), and *p* < 0.05 was considered statistically significant. 

This study was conducted with the approval of the Ethics Committee of the University of Tokyo School of Medicine (2018150NI).

## 3. Results

The questionnaires were distributed to 707 CMs at academic conferences and workshops. The recovery rate was 72.6%, with 513 participants. A total of 5 participants did not agree with the study and were therefore excluded, leaving a total of 508 participants. 

Descriptive statistics of the participants’ demographics and their experience in cases involving the spousal death of people with dementia are shown in Table 1. The participants were predominantly women, with 80% in their 50s. More than 70% had more than 10 years of experience as a CM, and about 80% were qualified as senior CMs. Over 80% of the participants had experienced the spousal death of a patient with dementia. However, the percentage of those who disclosed the death of the patient’s spouse was relatively evenly distributed, ranging from 0% to 100%. Furthermore, among those who did disclose the information, nearly 20% experienced a worsening of the patient’s BPSD as a result of the disclosure, and nearly 30% experienced a worsening depression in the patient with dementia. 

Descriptive statistics on participants’ attitudes and actual behaviors regarding disclosure are shown in Table 2. Regarding the attitude toward disclosure, “should be disclosed” and “better to disclose,” which are categorized in the pro-disclosure group, accounted for about 83% of the participants, and most of the subjects were positive about disclosure. Nearly half of the participants cited the person’s right to know and the family’s intention as priorities when considering the issue of disclosure, and about a quarter cited the person’s personality, the degree of progression of dementia, and the presence or absence of depression. When asked about the level of progression of dementia, over 40% of the respondents answered that the patient should be informed regardless of the level of progression, even if the patient is bedridden or unable to speak. However, the remaining nearly 60% thought that the level of dementia severity should be taken into account in deciding whether to disclose or not. When the patient’s family expressed the wish to not disclose, “non-intervention” and “reconsideration of disclosure” were selected by nearly 40% of the respondents. However, about 15% of the participants also responded “recommend disclosing” or “persuade to disclose.” Most of the respondents also consulted with other staff members, such as colleague CMs, the patient’s attending physician, and multi-professional conferences, but nearly 10% said the respondents never consulted with other staff members. 

Table 3 shows the consistency between the attitude toward disclosure and the disclosure rate for those who had experienced the spousal death of a patient with dementia. In the pro-disclosure group, about 44% were in the low disclosure rate group with an actual disclosure rate of 50% or less, while only about 17% were in the anti-disclosure group with an actual disclosure rate of 60% or more. The pro-disclosure group showed a larger discrepancy between the attitude toward disclosure and the actual disclosure rate.

Focusing only on the pro-disclosure group, Table 4 shows the results of a comparison between the low disclosure rate group (≤50%) and the high disclosure rate group (≥60%) in terms of participants’ demographics, experience with the spousal death of a patient with dementia, attitude toward disclosure, and actual behavior regarding disclosure. Statistically significant differences between the high and low disclosure rate groups in the pro-disclosure group were found in the percentages of participants who cited the patient’s personality, presence or absence of BPSD, and marital status as priorities when considering disclosure issues. Additionally, it includes the percentage of participants who said they would not consult with other staff if the family wished not to disclose the patient’s condition. The low disclosure rate group in the pro-disclosure group, i.e., those who thought that disclosure should be provided but did not disclose much, had a larger proportion of those who thought that the personality of the patient and the presence or absence of BPSD were high priorities and a smaller proportion of those who thought that marital relations were a high priority compared to those who disclosed more. In addition, the percentage of those who said that they do not consult with other staff when a family member wishes not to inform the patient was smaller in the low disclosure rate group than it was in the high disclosure rate group.

## 4. Discussion

The results of this study provide the first current understanding of CMs’ attitudes toward disclosure and actual behaviors for disclosing the death of a spouse to a patient with dementia in Japan. Although the event of the spousal death of patients with dementia is frequently experienced by CMs, the implementation of disclosure varies from case to case. Since a certain number of CMs have experienced a worsening of BPSD and depression in their patients due to disclosure, concern about the possibility of such worsening may have influenced the implementation of disclosures. In addition, as indicated by the high priority given to the family’s intention (51%) when considering the issue of disclosure, the family’s intention tends to be important in the care of patients with dementia in Japan, and the family’s desire not to inform the patient may have influenced the implementation of the disclosure.

In our study, 82.3% of our participants were female, compared with 70% of the results of the survey on the treatment status of long-term care workers conducted by the Ministry of Health, Labor, and Welfare (2018a). The age distribution of the participants was higher than that of the same survey. Approximately 90% of this study’s participants had acquired senior CM status, compared with 35% of the completion rate of the senior CM training program in the survey conducted by the Ministry on home care support offices and works of care managers (2018b). The ratio of certified CMs was 38.3%, which was considered to be a very high ratio for the acquisition rate of 0.2% nationwide. Therefore, the participants in this study were slightly older than average and had a high percentage of senior CMs and certified CMs.

Regarding disclosure, about 83% stated positive opinions about disclosure, meaning that their policies were in line with those defending the rights of people with dementia. Although this survey does not explore the meaning of such a belief, it may be speculated that it is based on the way of life of a person living through the death of his or her spouse. If we do not ask patients with dementia to pursue such an essential way of life, we would regard them as having already deviated from their original way of life. Thus, we would hesitate to judge them in this way.

In the pro-disclosure group, about 44% were in the low disclosure rate group with an actual disclosure rate of less than 50%, indicating that a relatively large number of CMs face the dilemma of “thinking it would be better to disclose, but not being able to do so given the patient’s condition.” The results on the relationship between BPSD and depression and disclosure showed that attitudes toward BPSD influenced the disclosure rate. This is because depression does not increase the caregiving effort. However, worsening BPSD increases the caregiving effort and may lead to risky behavior, making CMs who have experienced BPSD more cautious about disclosing it.

It is instructive that the percentage of those who stated that they would not consult with other staff if a family member wished not to inform the patient was smaller in the low disclosure rate group than it was in the high disclosure rate group. This may indicate that the CMs’ beliefs regarding the patients’ best interests are changing as a result of discussion with others. It is generally believed that when ethical issues arise in the field of care, it is useful to discuss them with a multi-professional team to resolve them [25]. Furthermore, it can be considered that the integration of the ideas of each individual through consultation with others results in a more valid conclusion. In this sense, it is necessary for CMs to properly consult with multiple professionals rather than taking actions toward disclosure based solely on their ideas. Even if the rate of disclosure is reduced as a result of this, it should be evaluated as an increase in the ethical validity in the medical field. However, there is a possibility that the respondents may have shortsightedly avoided responsibility by consulting with others, and further research is needed in the future.

The possible limitations of this study include, first, that sampling bias may not have been eliminated. The participants from this study varied in sex, age, qualification acquisition rate, and so on from the entire CM population. In addition, the participants for this study were recruited from the participants of conferences and workshops. CMs who attend academic conferences and workshops are considered to be a group that is eager to improve their skills and acquire new knowledge. However, many CMs in Japan do not attend academic conferences and workshops. So, this may not represent the results for CMs in Japan as a whole. Second, selection bias, which has many research subjects interested in dementia, and recollection bias, which is a survey of past experiences, may also be factors. In addition, since this study is an exploratory survey of the current situation in Japan, the validity of the questionnaire has not been fully examined, and other factors may be relevant. Third, this study did not examine the positive effects of the disclosure of spousal death for patients with dementia because it was outside the scope of the research question. It is possible that the disclosure of spousal death for patients with dementia may have positive effects that we did not examine. Despite these limitations, this study is the first fact-finding research work on the disclosure of spousal death for people with dementia, and this is the first time that CMs’ policies for disclosure and the disclosure rate were examined. In addition, although accurate figures cannot be given for worsening BPSD and depression associated with disclosure, the fact that the experience ratio of CMs was aggregated can be considered a strength. Having gained an understanding of the ethical dilemma situation in which CMs find themselves, this study provides a foundation for future studies. It will be necessary to further refine the questions asked in the interview survey and conduct a questionnaire survey of a more representative group. Simultaneously, however, this study is the first step toward social outcomes. It is hoped that professional groups, such as academic organizations to which caregivers belong, will consolidate their unified views on the issue of disclosure. This is expected to confront the care environment for people with dementia, including CMs, in the future, and efforts will be made to formulate a certain set of guidelines. This may lead to a more improved care environment, including CMs, which, in turn, may contribute to improving the well-being of people with dementia. This study can be used as a basis for this purpose.

## 5. Conclusions

This study is the first survey conducted on the disclosure of spousal deaths of patients with dementia. Although more than 80% of CMs had experienced the spousal death of patients with dementia, the percentage of disclosures varied widely. In Japan, disclosure likely reflects the wishes of the family. However, simultaneously, it may also be influenced by the worsening of BPSD experienced by about 18% and the worsening of depression experienced by 26% of the participants. Nearly 83% of the participants were positive about disclosure, but about 44% of them disclosed less than 50% of the time. This indicates that a relatively large number of caregivers may be in a dilemma regarding disclosure. Despite the limitation of sampling bias in that the participants were recruited from academic conferences and workshops, along with the validity of the questionnaire, we found the actual situation of CMs’ attitudes regarding the management of the disclosure of a spousal death for patients with dementia. It is expected that this study will be used as a significant basis for formulating guidelines to address the disclosure issues of the care environment for patients with dementia regarding care management. 

## Figures and Tables

**Table 1:** Basic attributes of the participants, and the experience with spousal deaths of patients with dementia.

			n (%)	
Attributes	Sex	Male	83	(16.3%)
		Female	385	(75.8%)
		No response	40	(7.9%)
	Age	≤30s	31	(6.1%)
		40s	140	(27.6%)
		50s	219	(43.1%)
		≥60s	91	(17.9%)
		No response	27	(5.3%)
	Years of experience as CM	<10 years	138	(27.2%)
		10–14 years	171	(33.7%)
		≥15 years	187	(36.8%)
		No response	12	(2.4%)
	Qualification of senior CM	No	110	(21.7%)
		Yes	398	(78.3%)
	Qualification of certified CM	No	338	(66.5%)
		Yes	170	(33.5%)
Experience	Experiences with spousal deaths of patients with dementia	0	81	(15.9%)
		1–2	128	(25.2%)
		3–5	171	(33.7%)
		≥6	114	(22.4%)
		No response	14	(2.8%)
	Disclosure rate (only for those with case experience)	0, 10, 20, 30%	116	(28.1%)
		40, 50, 60, 70%	126	(30.5%)
		80, 90, 100%	144	(34.9%)
		No response	27	(6.5%)
	Experienced problems with worsening BPSD due to disclosure (only for those who have experienced disclosure)	Yes	67	(18.4%)
		No	288	(78.9%)
		No response	10	(2.7%)
	Experienced problems with worsening depression due to disclosure (only for those who have experienced disclosure)	Yes	95	(26.0%)
		No	255	(69.9%)
		No response	15	(4.1%)

**Table 2:** Attitude toward disclosure and actual behavior regarding disclosure.

			n (%)	
Attitude	Attitude on disclosure	Should be disclosed	201	(39.6%)
		Better to disclose	219	(43.1%)
		No need to disclose	73	(14.4%)
		Better to not disclose	6	(1.2%)
		No response	9	(1.8%)
	Priorities when considering disclosure issues (multiple responses)	Patient’s right to know	245	(48.2%)
		Personality of the patient	126	(24.8%)
		Progression of dementia	127	(25.0%)
		Presence or absence of BPSD	118	(23.2%)
		Presence or absence of Depression	124	(24.4%)
		Marital relationship	102	(20.1%)
		Family structure	31	(6.1%)
		Family’s intention	259	(51.0%)
		Opinion of healthcare provider	27	(5.3%)
		Other	6	(1.2%)
		No answer	91	(17.9%)
	How far along in the progression of dementia do you think it is better to disclose?	To the extent of the progression that the patient’s cognitive function is age-appropriate, but the patients are forgetful	70	(13.8%)
		To the extent of the progression that the patient has no problems in daily life but cannot perform complex tasks	30	(5.9%)
		To the extent of the progression that the patient has trouble managing money or shopping	59	(11.6%)
		To the extent of the progression that the patient is no longer able to choose his/her own clothes	22	(4.3%)
		To the extent of the progression that the patient requires assistance in many daily activities	54	(10.6%)
		To the extent of the progression that the patient’s vocabulary and facial expressions have deteriorated and walking has become difficult	34	(6.7%)
		Regardless of the degree of progress, even if the patient is bedridden or unable to speak	218	(42.9%)
		No response	21	(4.1%)
Actual behavior	Action for family members if they wish not to disclose to the patient	Respect the family’ wishes as they are and do not intervene specifically	196	(38.6%)
		Talk to the family about the advantages and disadvantages of telling the person and ask them to reconsider	202	(39.8%)
		Respect the patient’s right to know and suggest to the family that they tell the patient, or try to persuade the family to tell the patient	78	(15.4%)
		No response	32	(6.3%)
	Actions for other staff if family members prefer not to disclose to the patient (multiple responses)	Consult with a colleague CM	262	(51.6%)
		Ask the attending physician of the patient for his/her opinion	204	(40.2%)
		Hold a multi-professional meeting	230	(45.3%)
		Basically, do not consult with other staff	42	(8.3%)
		No response	36	(7.1%)

**Table 3:** Consistency of the attitude toward disclosure and the disclosure rate among those who experienced the spousal death of a patient with dementia.

	Low Disclosure Rate (Disclosure Rate ≤ 50%)		High Disclosure Rate(Disclosure Rate ≥ 60%)	
Pro-disclosure	141	(43.8%)	181	(56.2%)
Anti-disclosure	49	(83.1%)	10	(16.9%)

**Table 4:** Difference between the low and high disclosure rate groups in the pro-disclosure group.

			Low Disclosure Rate Groups n (%)		High Disclosure Rate Groups n (%)		Fisher’s Exact Test *p*-Value
Attributes	Sex	Male	26	(19.7%)	30	(18.0%)	0.766
		Female	106	(80.3%)	137	(82.0%)	
	Age	≤30s	11	(8.4%)	10	(5.7%)	0.354
		40s	32	(24.4%)	57	(32.6%)	
		50s	59	(45.0%)	77	(44.0%)	
		≥60s	29	(22.1%)	31	(17.7%)	
	Years of experience as CM	<10 years	37	(26.6%)	38	(21.5%)	0.521
		10–14 years	45	(32.4%)	58	(32.8%)	
		≥15 years	57	(41.0%)	81	(45.8%)	
	Qualification of senior CM	No	24	(17.0%)	37	(20.4%)	0.476
		Yes	117	(83.0%)	144	(79.6%)	
	Qualification of certified CM	No	92	(65.2%)	110	(60.8%)	0.419
		Yes	49	(34.8%)	71	(39.2%)	
Experience	Experiences with spousal deaths of patients with dementia	1–2	46	(32.6%)	47	(26.0%)	0.317
		3–5	59	(41.8%)	76	(42.0%)	
		≥6	36	(25.5%)	58	(32.0%)	
	Experienced problems with worsening BPSD due to disclosure	Yes	28	(20.4%)	25	(14.0%)	0.171
		No	109	(79.6%)	153	(86.0%)	
	Experienced problems with worsening depression due to disclosure	Yes	36	(26.7%)	44	(25.0%)	0.794
		No	99	(73.3%)	132	(75.0%)	
Policy	Priorities when considering disclosure issues (multiple responses)	Patient’s right to know	71	(58.7%)	104	(68.4%)	0.101
		Personality of the patient	45	(37.2%)	33	(21.7%)	0.007
		Progression of dementia	34	(28.1%)	31	(20.4%)	0.154
		Presence or absence of BPSD	47	(38.8%)	26	(17.1%)	<0.001
		Presence or absence of Depression	32	(26.4%)	51	(33.6%)	0.234
		Marital relationship	24	(19.8%)	53	(34.9%)	0.007
		Family structure	10	(8.3%)	7	(4.6%)	0.313
		Family’s intention	74	(61.2%)	97	(63.8%)	0.706
		Opinion of healthcare provider	5	(4.1%)	14	(9.2%)	0.150
		Other	1	(0.8%)	4	(2.6%)	0.387
Actual behavior	Behavior taken by CM toward family members when family members wish not to disclose to the patient	Respect the family’ wishes as they are and do not intervene specifically	54	(40.0%)	61	(34.7%)	0.614
		Talk to the family about the advantages and disadvantages of telling the person and ask them to reconsider	58	(43.0%)	81	(46.0%)	
		Respect the patient’s right to know and suggest to the family that they tell the patient, or try to persuade the family to tell the patient	23	(17.0%)	34	(19.3%)	
	Behavior taken by CM toward other staff when family members wish not to disclose to the patient (multiple responses)	Consult with a colleague CM	73	(54.5%)	89	(51.7%)	0.646
		Ask the attending physician of the patient for his/her opinion	56	(41.8%)	78	(45.3%)	0.563
		Hold a multi-professional meeting	69	(51.5%)	83	(48.3%)	0.645
		Basically, do not consult with other staff	7	(5.2%)	21	(12.2%)	0.045

## Data Availability

The datasets analyzed in the current study are available from the corresponding author upon reasonable request.
